# Osteonevus of Nanta—A Rare Case Report of a Cellular Blue Nevus with Ossification

**DOI:** 10.3390/reports8030139

**Published:** 2025-08-06

**Authors:** Camilla Soendergaard Kristiansen, Anna Louise Norling, Birgitte Bols, Christian Lyngsaa Lang

**Affiliations:** 1 Department of Plastic Surgery and Burns Treatment, Copenhagen University Hospital, 2100 Copenhagen, Denmark; 2 Department of Plastic Surgery, Herlev Hospital, 2730 Herlev, Denmark; 3 Department of Pathology, Herlev Hospital, 2730 Herlev, Denmark

**Keywords:** osteonevus of Nanta, cellular blue nevus, melanocytic nevus, ossification

## Abstract

**Background and Clinical Significance**: Osteonevus of Nanta is a rare histological phenomenon characterized by bone formation within a benign melanocytic nevus, most commonly in intradermal nevi of the head and neck. Although osteonevus of Nanta is rare, ossification in a cellular blue nevus is even more uncommon. To date, only one case of a cellular blue nevus with ossification has been documented. This case report adds to the limited literature and emphasizes the clinical importance of recognizing this rare phenomenon, as osteonevus of Nanta has been potentially associated with malignant melanoma. **Case Presentation**: A 72-year-old woman presented with an asymptomatic, pigmented scalp lesion that had recently increased in size. On clinical examination, the tumor appeared as a well-demarcated, firm, and nodular mass with dark blueish to violet pigmentation that measured 15 × 12 × 7 mm. To ensure a definitive diagnosis and rule out malignancy, the lesion was excised with narrow margins. Histological examination revealed a cellular blue nevus with prominent osseous metaplasia. Due to the absence of clear margins, a wider re-excision was performed. No residual tumor was found, and the patient remained asymptomatic with no recurrence. **Conclusions**: This case represents only the second published example of a cellular blue nevus with ossification. While osteonevus of Nanta is benign, its potential association with malignant melanoma, as well as its clinical resemblance to malignant entities such as nodular melanoma, malignant blue nevus, and pigmented basal cell carcinoma, underscores the need for thorough clinical and histopathologic evaluation.

## 1. Introduction and Clinical Significance

Osteonevus of Nanta is a rare histological phenomenon characterized by the presence of bone formation within a benign melanocytic nevus [1,2]. This form of cutaneous osseus metaplasia is most commonly seen in intradermal nevi of the head and neck region, particularly in adult women, with an overall estimated incidence from 0.61% to 1.45% among pigmented skin lesions [3,4].

Although ossification in melanocytic lesions is already a seldom-encountered histological variant, its occurrence in a blue nevus is exceedingly uncommon. Only a few cases of so-called “blue osteonevus” have been documented since the first report by Baran and Civatte in 1959 [1,5,6].

In this case report, we present a 72-year-old woman with a pigmented nodular lesion on the scalp, where histological examination unexpectedly revealed an osteonevus of Nanta in a cellular blue nevus. To our knowledge, this is only the second published example of an ossified cellular blue nevus after the original description by Collina et al. in 1991 [6]. Although osteonevus of Nanta are benign and not associated with poor prognosis, it may occur in conjunction with malignant melanoma [7]. As such, it holds potential clinical relevance. Moreover, its clinical appearance may resemble malignant conditions such as nodular melanoma, malignant blue nevus, and pigmented basal cell carcinoma, further emphasizing the importance of thorough diagnostic evaluation. This report highlights the clinical, histological, and diagnostic aspects of this uncommon presentation, and briefly discusses its potential pathogenetic mechanisms.

## 2. Case Presentation

A 72-year-old woman presented with a skin lesion on the scalp of unknown exact duration. The patient had been aware of the lesion for many years but was unable to specify its duration more precisely. She was referred to our clinic as the lesion had recently increased in size, now measuring 15 × 12 × 7 mm. On clinical examination, the lesion appeared as a well-demarcated, firm, nodular tumor with dark blueish to violet pigmentation. She reported no pain, itching, or bleeding. The lesion had not previously undergone any treatment. Her medical history included hypertension and hypercholesterolemia, with no other known comorbidities and no personal or family history of melanoma or atypical nevi.

**Figure 1:**
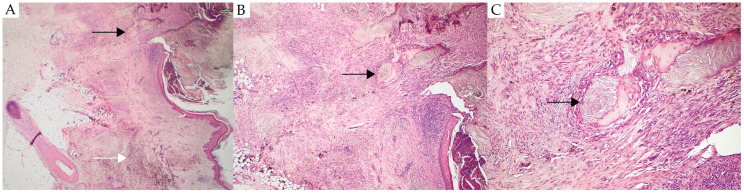
Histopathological features of an osteonevus of Nanta. (**A**) Low-power view showing skin and subcutaneous tissue. The black arrow highlights an area of bone formation surrounded by blue nevus cells. The white arrow highlights pigmented melanocytes in the dermis, consistent with a cellular blue nevus. (**B**) Medium-power view (×4) of the same area, with the black arrow indicating bone formation surrounded by blue nevus cells. (**C**) High-power view (×10) of the same area, again with the black arrow indicating bone formation surrounded by blue nevus cells.

Due to the lesion’s pronounced dark pigmentation and nodular appearance, differential diagnoses included angioma, benign melanocytic nevus, malignant blue nevus, seborrheic keratosis, pigmented dermatofibroma, nodular melanoma, and pigmented basal cell carcinoma. To ensure a definitive diagnosis and rule out malignancy, the lesion was excised with narrow margins. The lesion was not examined with dermoscopy prior to excision. Histopathological examination unexpectedly revealed a cellular blue nevus with ossification, also known as osteonevus of Nanta, or a so-called “blue osteonevus” (Figure 1). The epidermis exhibited both an exophytic process and, in certain areas, a more endophytic growth pattern. A central area of ulceration was also observed, with corresponding marked bone formation. No atypia was observed. There was no melanocytic component in the epidermis. In the dermis, extending into the subcutis, bundles of spindle-shaped cells with marked neurotropic differentiation were observed. The cells were predominantly uniform, with small, round to slightly oval nuclei, although areas with more irregular nuclei were also noted. Pigment deposits and melanophages were present in certain areas. Immunohistochemical staining for Ki-67 showed no overexpression. The melanocytic nature of the lesion was confirmed by strong immunopositivity for SOX10, S-100, and Melan-A.

As osteonevus of Nanta has been reported in association with malignant melanoma, the tumor was re-excised with wider margins of 5 mm due to the absence of clear surgical margins in the initial excision. Follow-up histological analysis showed no residual tumor, and the patient experienced no symptoms or recurrence during a three-year follow-up period.

## 3. Discussion

Osteonevus of Nanta represents a rare histological phenomenon, with osseous metaplasia occurring within benign melanocytic nevi, most commonly of the intradermal type [2]. The phenomenon was first described in 1908 by Heidingsfeld, who reported ossification within a melanocytic nevus. However, it was more thoroughly characterized and formally published by Nanta in 1911, leading to the term “Osteonevus of Nanta” [8].

In the literature, osteonevus of Nanta is most commonly reported in the head and neck region, with variable age of presentation, though it is most frequently observed in adults. While most studies have found a higher incidence in females [8,9], Bezić et al. [2] reported a nearly equal distribution between males and females. The location, age, and sex of the patient in our case are therefore consistent with previously published findings.

The pathogenesis is not fully understood, but several theories have been proposed. One of the main theories proposed to explain ossification in melanocytic nevi is the hamartomatous (disembryoplastic) hypothesis. This theory suggests that the presence of mesenchymal stem cells with osteogenic potential at ectopic sites ultimately leads to this type of hamartomatous malformation with the coexistence of tissues of both ectodermal and mesodermal origin within the same lesion [8].

Another leading theory proposes that ossification arises through osseous metaplasia, triggered by repeated trauma, chronic inflammation, or proliferating melanocytes [8,10]. These factors are thought to induce the differentiation of dermal fibroblasts into osteoblasts. The inflammatory response is often attributed to follicular rupture, which may result from physical distortion or obstruction caused by melanocyte accumulation. In some cases, repeated trauma, such as hair plucking or shaving of pigmented nevi, can damage hair follicles and initiate a localized inflammatory cascade, ultimately promoting bone formation [1,8].

A third theory suggests a potential role for estrogen in bone formation, as osteoblasts are known to express surface receptors for estrogen [2]. This has been proposed to explain the higher incidence of osteonevus of Nanta in adult females. In our case, immunohistochemical staining for estrogen receptors was not performed, but this could have been of interest to explore in light of the theory suggesting a role for estrogen in bone formation associated with osteonevus of Nanta.

In the present case, none of the three proposed mechanisms can be definitively confirmed, yet each holds varying degrees of plausibility. The hamartomatous theory remains a foundational explanation, as the coexistence of melanocytic and osseous elements may reflect an embryologic anomaly. The trauma and inflammation-induced theory appears less likely in our case. The lesion was located on the scalp, where repetitive mechanical irritation, such as hair plucking or shaving, is generally uncommon in elderly women, and the patient denied any such behaviors or symptoms including scratching.

The hormonal theory, suggesting a role for estrogen in bone formation, may be relevant given the patient’s sex. However, her postmenopausal status would be associated with lower estrogen levels. Furthermore, a case reported by Tartaglia et al. [10] describing osteonevus of Nanta in a prepubertal male child suggests that estrogen receptor activation is not a necessary factor in the ossification of melanocytic nevi.

Taken together, the hamartomatous origin appears most consistent with the clinical and histopathological findings in our patient.

Although osteonevus of Nanta in an intradermal nevus is a rare finding, ossification within a blue nevus, referred to as a blue osteonevus, is even more uncommon [1]. Since the initial report by Baran and Civatte [5] in 1959 and the more specific case of a cellular blue osteonevus described by Collina et al. [6] in 1991, only one additional case has been documented in the literature [1]. To our knowledge, the present report represents only the second published case of a cellular blue nevus with osseous metaplasia.

Given the pigmented, nodular appearance and scalp location of the lesion, important differential diagnoses prior to excision included malignant blue nevus, nodular melanoma, and pigmented basal cell carcinoma.

Cellular blue nevi are generally benign, but their features can lead to diagnostic confusion with malignant blue nevus due to overlapping dermoscopic characteristics such as whitish scar-like depigmentation, dots or globules, peripheral streaks, and vascular structures [11,12]. This overlap can complicate clinical evaluation and reduce the specificity of dermoscopy in such lesions.

Nodular melanoma also represents a critical diagnostic pitfall, as it often presents as a well-circumscribed, symmetrically pigmented nodule without an apparent radial growth phase [13]. Clinically, it may resemble benign lesions such as blue nevi or vascular tumors, especially when deeply pigmented. In our case, the dark blue to violet coloration, firm consistency, and recent growth of the lesion could easily raise suspicion of nodular melanoma.

Pigmented basal cell carcinoma can mimic melanocytic lesions clinically, particularly when presenting as a pigmented, nodular lesion on sun-exposed areas like the scalp. It may appear as a blue-gray papule with a smooth or shiny surface, occasionally showing central ulceration or telangiectasia [14]. These features overlap with our patient’s lesion, which was also nodular, pigmented, and located on the scalp, reinforcing the clinical uncertainty.

These overlaps in clinical features highlight the importance of complete excision and histopathological evaluation to differentiate benign entities such as osteonevus of Nanta from malignancies. Furthermore, while osteonevus of Nanta is histologically benign, it has been associated with malignant melanoma [7]. Its occurrence with ulceration or signs of active growth, as seen in our case, warrants clinical caution. Although ulceration is a nonspecific finding, it may reflect rapid tumor growth and should not be overlooked [15].

Although dermoscopy may assist in distinguishing benign from malignant lesions, it cannot replace histological examination as the definitive diagnostic tool. In lesions with atypical pigmentation, nodularity, or recent growth, such as in our case, surgical excision remains essential for establishing an accurate diagnosis and guiding appropriate management.

Due to the absence of clear margins in the initial excision, the lesion was re-excised with wider surgical margins to exclude any residual or malignant components. Follow-up histological evaluation confirmed complete removal with no evidence of residual tumor. The patient remained asymptomatic and showed no signs of recurrence.

## 4. Conclusions

This case adds to the limited literature on the rare histopathologic phenomenon of a cellular blue nevus with ossification and highlights the importance of considering osseous metaplasia in the differential diagnosis of pigmented skin lesions. Although osteonevus of Nanta is generally regarded as benign and not typically associated with poor prognosis, its potential association with malignant melanoma warrants thorough clinical and histopathological evaluation. Moreover, the clinical presentation may closely mimic malignant conditions such as nodular melanoma, malignant blue nevus, and pigmented basal cell carcinoma, further emphasizing the need for complete excision and microscopic analysis to ensure accurate diagnosis. Further studies are needed to clarify the nature and frequency of this association.

## Data Availability

The original contributions presented in this study are included in the article. Further inquiries can be directed to the corresponding author.
